# Decomposition of gender differences in cognitive functioning: National Survey of the Japanese elderly

**DOI:** 10.1186/s12877-020-01990-1

**Published:** 2021-01-10

**Authors:** Shohei Okamoto, Erika Kobayashi, Hiroshi Murayama, Jersey Liang, Taro Fukaya, Shoji Shinkai

**Affiliations:** 1 Tokyo Metropolitan Institute of Gerontology, 35-2 Sakaecho, Itabashi-ku, Tokyo 173-0015 Japan; 2 The University of Tokyo, 7-3-1 Hongo, Bunkyo-ku, Tokyo 113-8656 Japan; 3 University of Michigan, 1415 Washington Heights, SPH II M3007, Ann Arbor, MI 48109-2029 USA; 4 Kagawa Nutrition University, 3-9-21 Chiyoda, Sakado City, Saitama 350-0288 Japan

**Keywords:** Aging, Dementia, Gender, Health, Socioeconomic status

## Abstract

**Background:**

It is well known that females generally live longer than males, but women tend to suffer from more illnesses and limitations than men do, also for dementia. However, limited empirical evidence is available why this ‘male-female health-survival paradox’ is observed. This study aimed to investigate factors which account for gender differences in health, particularly cognitive functioning and decline among older adults.

**Methods:**

Data were retrieved from the National Survey of the Japanese Elderly, which is a longitudinal survey of a nationwide representative sample of Japanese adults aged 60 or over. Gender differences in cognitive functioning and decline in three-year follow-ups were decomposed using Blinder–Oaxaca decomposition analysis, regarding demographic, socioeconomic, and health-related factors into the ‘explained’ component, by differences in individual attributes listed above, and the ‘unexplained’ component.

**Results:**

Empirical analyses showed that women’s lower cognitive functioning was partly explained by the endowment effect. Moreover, a shorter duration of formal education and a larger proportion with their longest occupation being domestic worker accounted for steeper cognitive decline and more prevalent mild cognitive impairment in women than in men.

**Conclusion:**

This empirical study suggested that gender differences in cognitive functioning and decline account for different individual attributes of social determinants among men and women. Particularly, men seem to be more engaged in activities which accumulate intellectual experiences through education and occupation, as suggested by the cognitive reserve hypothesis.

**Supplementary Information:**

The online version contains supplementary material available at 10.1186/s12877-020-01990-1.

## Background

### Male–female health differences

Dementia is a global issue that imposes increasing burdens on society as the ageing of the population progresses. Worldwide, Japan is one of the most ‘aged societies’, with 28.3% of the population reported to be aged 65 or over in 2019 [1]. Japan’s prevalence of dementia is estimated to be the highest among OECD countries [2], and is 15% among the population aged 65 or over [3]. Moreover, the estimated social cost of dementia in Japan is 14.5 trillion JPY (13 billion USD), close to 3% of the GDP [4]. As an effective cure for dementia is not yet available, it is important to incorporate various approaches, including changes in social policy, in the prevention of cognitive decline due to Alzheimer’s disease and other forms of dementia, particularly through improvements in education, social relationships, physical and psychological health, and the environment [5].

One frequently asked question is why men and women often have different health statuses. It is well known that females generally live longer than males, a pattern which also seems to prevail among other animal species [6]. Moreover, women are less likely to die from most major cause-specific diseases (e.g. heart diseases, cancer, and diabetes). However—in what might be a phenomenon unique to humans—women tend to suffer from more physical illnesses and limitations than men do, a phenomenon known as the *health–survival paradox* [6]. This paradox may be caused by mortality selection among men (i.e. men with severe health issues die earlier) or differences in types of health issues prevalent among men and women.

Male–female health differences seem to hold true for dementia. More women suffer from Alzheimer’s disease or other types of dementia than men in almost all age groups in Japan [3, 7], and many other regions [8–12]. This is potentially due to various factors, of which men and women have different distributions, affect cognitive functioning in both positive and negative ways [13–19]. Therefore, exploring sex/gender differences in health attributes has important implications for improvement in current health statuses and future health outcomes, by clarifying preventable risk factors which could be mitigated by policy changes. In the following sections, we review the potential mechanisms underlying sex/gender differences in cognitive impairment risks, focusing on behavioural risks and social determinants.

### Potential mechanisms in gender differences in cognitive impairment

Although there has been accumulating evidence recently regarding sex/gender-based health differences, this topic has not yet been fully understood [14] partly because of the underrepresentation of female participants in health and medical studies conducted to increase understanding of disease mechanisms [20]. Sex/gender differences in cognitive impairment have been approached considering both biological and social factors: While biological factors (e.g. effects of apolipoprotein E *ϵ* 4 genotype and gonadal hormones) can cause sex differences in health, particularly cognitive impairment, behavioural risk factors and social determinants also seem important in explaining gender differences [13–18, 21–25].

### Behavioural factors

Acquired risks and health behaviours may account for gender differences in cognitive impairment. As cardiovascular risks are associated with the onset of dementia [26, 27], health behaviours which influence these risks can play mediating roles. In fact, health behaviours such as exercise, smoking habits, and diet, have been associated with the risk of cognitive impairment [28–30]. Previous studies suggested that behavioural determinants are more significant in men [23], and men tend to be more engaged in risk behaviours for chronic conditions, while women are more likely to engage in preventive behaviours, such as keeping a healthy diet, not smoking, and accessing health services [14, 19, 21, 22].

Different susceptibilities and responses to another risk factor for dementia, psychological distress, may also account for gender-based risk differences. Women’s higher risk of depression [15] and psychological distress through gendered stressors and resources [31] could contribute to gender disparities in cognitive impairment while different stress coping styles between men and women were observed: Women tend to engage in a ‘tend-and-befriend’ reaction that promotes safety and reduces distress, more likely seeking social support than men do [21, 32, 33].

### Social determinants

Previous research has suggested that gender-based health differences can originate from social determinants (e.g. education, occupation, and economic conditions) [13–15, 17, 21–23]. Socioeconomic inequality among men and women is rooted in many factors, such as economic, political, historical, and social arrangements, and affects health and wellbeing via health-related mediators (e.g. social, institutional, and psychosocial factors and health behaviours), which are related to behavioural factors [22, 34]. From the perspective of life-course approach, even biological factors are strongly affected by exposure to adverse circumstances during critical developmental periods, such as in utero, infancy, and early childhood [35]. Furthermore, socioeconomic disadvantage measured by education is indirectly associated with an increased risk of dementia via psychological distress [36].

An important hypothesis used to explain gender differences in cognitive functioning from the viewpoint of social determinants is the cognitive reserve hypothesis. The cognitive reserve hypothesis, which suggests that intellectually stimulating activities protect against neurodegenerative changes through neural reserve and neural compensation, was proposed to interpret the phenomena of brain pathology not associated with clinical symptoms [37]. The author suggests that anatomical measures (e.g. brain size, head circumference, synaptic count, and dendritic branching), which are effective measures, and socioeconomic status (e.g. education and occupation), which is relatively easy to obtain, are widely used as proxies for reserve. It suggests that lifetime intellectual activities contribute to resistance to brain damage and disruption. It has been repeatedly reported that there is a negative association between education, regarded as one of the markers of measures of reserve, and both dementia and cognitive decline (e.g. X Meng and C D’Arcy [38]; also in Japan: S Okamoto [39]).

### Literature review

Many studies have observed male–female differences in the incidence, prevalence, and conditions of health issues and various hypotheses which potentially explain gender differences in cognitive impairment; however, there has not been sufficient empirical examination that directly investigates causes of gender differences in a representative sample. Previous works regarding gender differences in health assessed coefficients of gender variables or its interaction with factors—with or without multivariate adjustments—or compared variables which potentially explained gender differences after sex-specific analyses in health [36, 40–45]. However, these studies did not clarify whether gender-based health differences can be explained by endowments (i.e. distributions of explanatory variables for individual attributes, such as education, occupation, and health behaviours) or other effects (e.g. effects of the same attributes on health and other unexplained factors, such as biological differences). To our knowledge, only few studies evaluated whether gender differences in cognitive impairment were mitigated by social determinants [36, 44, 46]. However, these studies also did not distinguish between endowment effects and other effects.

Therefore, the present study aimed to investigate what accounts for gender differences in cognitive functioning among older Japanese adults, utilising an econometric method to decompose gender differences in cognitive functioning into endowments and other unexplained factors, from the viewpoint of behavioural risks and social determinants differences. Exploring factors which account for gender-based health inequality is helpful in obtaining future expectations for dementia prevalence and insights into risk reduction through policy interventions. We expected that higher reserve among men would contribute to higher cognitive functioning for men than women; however, this benefit for men may diminish due to poor health behavioural choices and health status, as suggested by our literature review.

## Method

### Data

Data were retrieved from the National Survey of the Japanese Elderly (NSJE), a nationally representative sample of Japanese adults aged 60 years or above. This survey started in 1987, with a sample (*n* = 2200) extracted from the Basic Resident Registration System, using a stratified two-stage random sampling method. It was subsequently supplemented with new samples in wave 2 (1990, *n* = 580), wave 4 (1996, *n* = 1210), and wave 5 (1999, *n* = 2000). Face-to-face interviews were performed during each wave, and participants received follow-up approximately once every 3 years.

We performed two types of analyses. First, we conducted survival analysis to assess factors associated with cognitive impairment using longitudinal data from wave 1(1987) to wave 8 (2012). Second, we performed decomposition analysis of gender differences using the first two waves (i.e. baseline and follow-up surveys of four groups: wave 1–2, wave 2–3, wave 4–5, and wave 5–6) of the newly added samples in waves 1, 2, 4, and 5, to minimise potential bias, which can be induced by differences in survey intervals or by competing risk factors (i.e. death) between men and women.

### Cognitive functioning

In the survey, cognitive functioning was measured by nine items, based on the Short Portable Mental Status Questionnaire (SPMSQ) [47, 48]. The questionnaire contained nine items: respondent’s home address, interview date, interview day, mother’s maiden name, name of the current prime minister, name of the previous prime minister, a simple calculation, respondent’s birthday, and respondent’s age.

Cognition was defined in three ways in this study: 1. number of incorrect answers at baseline (including cases in which a respondent did not know the answer), 2. number of increased incorrect answers between two waves (cognitive decline: marked zero if cognitive functioning improved between waves), and 3. cognitive impairment (mild impairment), ascertained by individuals with three or more incorrect answers [47, 48] at the second survey.

Utilising item response theory, we reviewed whether each item of the SPMSQ appropriately identified cognitive functioning. As shown in Additional file 1, the response to one of the items, ‘mother’s maiden name’, seemed to poorly identify cognitive functioning. Therefore, we checked the robustness of the results using eight items of the SPMSQ without this item, when cognitive functioning was the outcome. As its cut-off point may change after excluding some items, we only consider the level of cognitive functioning at baseline and the changes between two waves for eight items of the SPMSQ, excluding ‘mother’s maiden name.’

### Independent variables

Independent variables used for estimations were based on the baseline survey, to explain gender differences in cognitive functioning from the viewpoint of social determinants, health behaviours, and health. The variables were age, marital status, home ownership, education, employment status, longest-held occupation, participation in group activities, smoking, exercise, alcohol consumption, and chronic conditions related to dementia. The definition of each variable and rationale for using them are described below.

Marital status was dichotomised as whether a respondent was single (including bereavement and divorce) or not. Previous studies suggested that living alone increased the risk of cognitive decline, compared with those who are married or live with someone [49]. Home ownership was used as a proxy of economic status.

As for educational attainment, as one marker of cognitive reserve, years of education that respondents received were used. We also included its square to consider the non-linear relationship between years of education and cognitive functioning.

Employment status was a binary variable marked as ‘1’ if a respondent was currently in paid work, and ‘0’ otherwise. Studies in Japan have verified that employment among older adults has protective effects against cognitive decline, and retirement leads to worse cognitive functioning outcomes [50].

Longest-held occupation, another marker of cognitive reserve, was defined by five occupational categories: professional (reference), administrative/sales, manual labour, agriculture/forestry/fishery, and domestic worker.

Participation in group activities, an indicator of social integration related to health [51], was defined as a binary variable marked as ‘1’ if a respondent belonged to any type of group (e.g. neighbourhood association, senior citizen club, or hobby club), and ‘0’ otherwise.

Health behaviours, as behavioural risks of cognitive impairment discussed in Section 1.2, were measured by three variables: cigarette smoking, alcohol consumption, and exercise habits. Cigarette smoking was dichotomised as current smoker or non-smoker. Alcohol consumption was a binary variable coded as ‘1’ if a respondent drinks alcohol, and ‘0’ otherwise. Exercise habits were recorded as a binary variable marked as ‘1’ if a respondent reported they often or sometime exercised, and ‘0’ if they hardly or never exercised.

We included four health conditions (hearing impairment, diabetes mellitus, hypertension, and stroke) as factors that explained cognitive functioning and decline [26, 27, 52]. Hearing impairment was dichotomised as respondents who wear hearing-aids or who reported any difficulty in hearing. Morbidities correspond to cardiovascular risk factors associated with the risk of cognitive decline and dementia.

To take period effects (e.g. in policy, economy, and technology) into account, we controlled for the wave in which an individual participated in the survey.

### Empirical approach

#### Factors associated with cognitive impairment in waves 1–8

First, we analysed longitudinal data for, at most, 25 years from wave 1 (1987) to wave 8 (2012), to verify factors associated with mild impairment (three or more incorrect answers of nine questions) and whether social determinants, behavioural risks, and health conditions mitigated the association between gender and cognitive impairment. We implement competing-risks regression [53] to obtain sub-hazard ratios of cognitive decline, adjusting for competing risks (i.e. death without an observation of cognitive impairment). Information on death, including date, was primarily obtained through residential records. If the death date was not available, we approximated it using the intermediate point between a wave when the death was ascertained and the previous wave (*n*=37). A total of 4329 individuals (1958 men and 2371 women) who responded to questions by themselves at least once in eight waves, and who had the necessary information, were analysed in this preliminary framework.

However, decomposition analysis for competing-risks regression was not performed, since biases for decomposition analysis for panel data and survival outcome data could be problematic [54, 55]. Thus, this analysis did not distinguish between endowment effects and other effects, similar to previous works [40–44]. Therefore, decomposition analyses for the first two waves were performed as discussed in the next section, for a better understanding of gender differences in cognitive functioning and decline.

### Decomposition analysis of gender differences

To assess factors which could account for gender differences in cognitive functioning and decline, we analysed NSJE data between the first two waves, since interval length was the same (3 years) in whichever wave the samples were added. Longer follow-up intervals may be more advantageous to observe distinct cognitive decline, since basic cognitive functioning declines with age [56]. However, sample attrition due to severe health problems, including death and dementia, may become more frequent in later waves; therefore, selection bias becomes more of a concern. Moreover, intervals among waves were not equal after wave 6, which may generate differences in observed outcomes or loss to follow-up. Thus, we used information from the first two waves of three-year follow-up of the newly added samples in waves 1, 2, 4, and 5 for the decomposition analyses.

We decomposed gender differences in cognitive functioning using the Blinder–Oaxaca decomposition method [57, 58]. This method was originally developed for labour economics, to decompose average wage differences among genders or races into *endowment effects*, which denote distributions of individual attributes, and *residual effects* which denote other factors, including discrimination. This decomposition method is beneficial in detecting what accounts for gender differences in cognitive functioning and decline, even when overall gender differences are not observed as one factor offsets another. Some previous studies made limited assessments of gender differences in health, since they compared the magnitude of a coefficient of a gender-dummy variable (e.g. a variable that is assigned a value of one when a respondent is female) with or without control variables, or of coefficients of covariates obtained from gender-specific analysis [40–44].

In this study, we estimated the following equation:

$$ \overline{Cog_M}-\overline{Cog_F}=\sum {\overline{X}}_M{\beta}_M-\sum {\overline{X}}_F{\beta}_F=\sum \left({\overline{X}}_M-{\overline{X}}_F\right){\beta}^{\ast }+\left[\sum {\overline{X}}_M\left({\beta}_M-{\beta}^{\ast}\right)+\sum {\overline{X}}_F\left({\beta}^{\ast }-{\beta}_F\right)\right], $$

where M and F stand for estimates in men and women, respectively. $\overline{\mathrm{Cog}}$ denotes the group-specific mean of cognitive functioning, *X* is a vector which includes independent variables and a constant, and β is a parameter. β^∗^ is a non-discriminatory parameter vector obtained from the whole sample. Thus, the mean outcome difference in cognitive functioning between men and women is decomposed into the ‘explained’ component (endowment), or the cognitive functioning/decline differential explained by gender differences in independent variables, and the ‘unexplained’ component, which also captures all potential effects of unobserved variables. Here, we analysed three different outcomes of the number of incorrect answers at baseline, the number of increased incorrect answers between two waves, and mild impairment, described in Section 2.2, using nine items of the SPMSQ.

Table 1 shows descriptive statistics by gender. Women had more incorrect answers than men at baseline and the second wave (3 years later), which implied men showed higher cognitive functioning than women, on average. Men were likely to have a longer education than women. Differences between men and women in longest occupation, proportion of current workers, single individuals, and smokers were also remarkable. Based on educational level and longest professional occupation, men seemed to generally have higher cognitive reserve than women.

**Table 1:** Descriptive statistics

	Men (*n*=1562)		Women (*n*=2019)		*P*-value^b^
	Mean/Proportion	SD^a^	Mean/Proportion	SD	
Memory mistakes [t=baseline]	0.62	0.93	0.86	1.03	< 0.001
Memory mistakes [t+ 1]	0.94	1.24	1.11	1.26	< 0.001
Memory mistakes [t+ 1]-[t]	0.58	0.99	0.58	0.98	0.916
Mild decline [t+ 1]	9.9%		12.8%		< 0.01
Moderate decline [t+ 1]	2.0%		2.0%		0.970
Age	68.02	6.53	68.66	6.77	< 0.001
Years of education	9.80	2.82	8.79	2.32	< 0.001
Current worker	48.7%		25.8%		< 0.001
Longest occupation					
Professional	23.0%		5.4%		< 0.001
Clerical	16.4%		26.4%		
Manual	40.3%		17.1%		
Agriculture/forestry/fishery	18.2%		10.8%		
Domestic worker	2.0%		40.2%		
Single	9.7%		47.1%		< 0.001
Home ownership	89.6%		86.4%		< 0.01
Group activity	68.6%		63.9%		< 0.01
Current smoker	45.5%		7.2%		< 0.001
Alcohol consumption	64.9%		7.2%		< 0.001
Exercise	52.7%		48.0%		< 0.01
Hearing impairment	6.6%		4.2		< 0.001
Diabetes	7.2%		4.4%		< 0.001
Hypertension	25.7%		30.8%		< 0.001
Stroke	3.9%		3.0%		0.125
Entry wave					
Wave1	45.9%		45.5%		
Wave2	8.7%		8.0%		
Wave4	20.8%		18.6%		
Wave5	24.6%		27.8%		

^a^*SD* standard deviation

^b^Welch’s method to test the difference of averages under the hypothesis of heteroskedasticity. Chi-square test was used for assessing differences of categorical variables between men and women

**Fig. 1:**
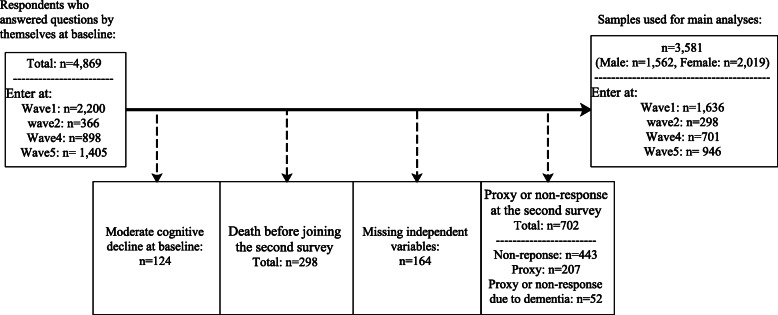
A flow chart for sample size calculation

Fig. 1 shows the procedure for calculating our sample size for decomposition analyses. A total of 4869 participants responded to the baseline survey by themselves. Those who marked a low percentage of correct answers at baseline (four correct answers or less out of nine), defined as ‘moderate decline’ by E Pfeiffer [47], were removed, as the accuracy of their responses can be low (*n* = 124). Additionally, those died before the second survey were excluded (*n*=298). Individuals without necessary information regarding independent variables (*n* = 164), and those who did not respond to the second survey by themselves (*n*=702) were also excluded from the analyses. Thus, the final sample size comprised 3581 individuals (1562 men and 2019 women).

## Results

### Factors associated with cognitive impairment in waves 1–8

Descriptive statistics regarding the outcome of competing-risks regression are shown in Additional file 1. About 29.2% of men and 38.9% of women were observed to experience cognitive impairment. About 45.6% of men and 29.1% of women died without an observation of cognitive impairment (competing-risk) before follow-up.

Table 2 shows the results of competing-risks regression by gender. For both men and women, higher educational attainment was significantly associated with lower risk of cognitive impairment: sub-hazard ratio (SHR), 0.827 (95% confidence intervals obtained by robust standard error [CI]: 0.721–0.950) for men and SHR: 0.800 (95%CI: 0.724–0.884) for women.

**Table 2:** Results of sex-specific competing-risks regression ^a, b, c, d^

	Men	Women
Age	0.995	1.014
	(0.956–1.036)	(0.985–1.044)
Age^2^/100	1.000	1.000
	(1.000–1.000)	(1.000–1.000)
Years of education	0.827**	0.800**
	(0.721–0.950)	(0.724–0.884)
Years of education^2^/100	1.675	1.785#
	(0.823–3.407)	(0.948–3.362)
Current worker	0.980	1.010
	(0.943–1.018)	(0.972–1.049)
**Longest occupation (Ref. Professional)**		
Clerical	1.171	1.313
	(0.850–1.614)	(0.862–1.999)
Manual	1.516**	1.619*
	(1.142–2.013)	(1.048–2.501)
Agriculture/forestry/fishery	1.336#	1.811**
	(0.967–1.845)	(1.156–2.837)
Domestic worker	2.127**	1.697*
	(1.199–3.773)	(1.118–2.575)
Single	0.966	1.007
	(0.909–1.026)	(0.977–1.038)
Home ownership	1.037	0.980
	(0.978–1.100)	(0.944–1.018)
Group activity	0.970	1.015
	(0.935–1.007)	(0.987–1.045)
Current smoker	0.979	1.017
	(0.944–1.014)	(0.969–1.067)
Alcohol consumption	1.011	0.958*
	(0.975–1.049)	(0.925–0.993)
Exercise	0.989	0.999
	(0.954–1.024)	(0.972–1.026)
Hearing impairment	0.971	1.045
	(0.906–1.040)	(0.981–1.112)
Diabetes	1.005	0.951
	(0.926–1.090)	(0.870–1.040)
Hypertension	0.992	0.977
	(0.953–1.033)	(0.948–1.007)
Stroke	0.989	1.029
	(0.888–1.103)	(0.919–1.151)
Observations	1958	2371

^a^ Sub-hazard ratio with 95% confidence intervals obtained by robust standard errors in parentheses

^b^ ** *p*< 0.01, * *p*< 0.05, # *p*< 0.10

^c^ Participating waves are included in the models

^d^ Age, working status, being single, home ownership, group activity, and health-related variables are treated as time-varying covariates by multiplying the logarithm of analysis time

The association between longest occupation and cognitive impairment was also found to be significant, in similar ways for men and women. Those engaged in manual labour (SHR: 1.516 [95%CI: 1.142–2.013] for men and SHR: 1.619 [95%CI: 1.048–2.501] for women) and domestic work (SHR: 2.127 [95%CI: 1.199–3.773] for men and SHR: 1.697 [95%CI: 1.118–2.575] for women) tended to experience more cognitive impairment than those in professional jobs. These results support the cognitive reserve hypothesis that education and occupation, which can be regarded as markers of measures of reserve, affect cognitive functioning later in life.

Table 3 compares the risk of cognitive decline for women, in reference to men, from four different models with or without social determinants and/or health-related variables. Compared to the null model, which only included age and participating waves (model 1), both health-related variables and social determinants seemed to mitigate gender differences in cognitive impairment (models 2 to 4). Social determinants can have larger effects on gender differences in cognitive impairment than the health-related variables used in this study.

**Table 3:** Results of competing-risks regression: Model comparison^a, b, c, d^

	Model 1	Model 2	Model 3	Model 4
Women	1.438**	1.365**	1.241**	1.172*
	(1.294–1.597)	(1.204–1.547)	(1.081–1.424)	(1.004–1.369)
Health	No	Yes	No	Yes
Social determinants	No	No	Yes	Yes
Age+ Participating waves	Yes	Yes	Yes	Yes
AIC	23,397	23,401	23,242	23,249
Observations	4329			

^a^ Sub-hazard ratios with 95% confidence intervals obtained by robust standard errors in parentheses

^b^ ** *p*< 0.01, * *p*< 0.05, # *p*< 0.10

^c^ Age, working status, being single, home ownership, group activity, and health-related variables are treated as time-varying covariates by multiplying the logarithm of analysis time

^d^ The full table is available upon request

### Decomposition analysis of gender differences

Table 4 shows the results of decomposition analysis of gender differences in cognitive functioning, cognitive decline, and cognitive impairment. On average, cognitive functioning for women was lower than for men at baseline, and approximately half the differences were ‘explained’ by differences in independent variables, while the remaining half were ‘unexplained’, which could potentially include biological factors.

**Table 4:** Decomposition of risk factors: the nine SQMSQ items^a, b, c, d^

	Cognitive functioning: No. of incorrect answers [t=baseline]		Cognitive decline: Increase in the no. of incorrect answers [t+ 1]-[t]		Cognitive impairment	
Male	0.622 (0.024)		0.577 (0.025)		1.104 (1.088–1.121)	
Women	0.857 (0.023)		0.581 (0.022)		1.137 (1.121–1.153)	
Difference	− 0.235**(0.033)		− 0.004 (0.033)		0.971**(0.952–0.991)	
	Explained	Unexplained	Explained	Unexplained	Explained	Unexplained
Overall	−0.116**(0.036)	−0.119*(0.046)	− 0.124**(0.035)	0.121**(0.047)	0.949**(0.922–0.976)	1.024 (0.987–1.062)
Age	0.046 (0.038)	2.975 (7.742)	0.047 (0.041)	−15.775*(7.764)	1.002 (0.981–1.024)	0.034 (0.000–3.964)
Age^2^/100	− 0.057 (0.040)	−1.422 (3.791)	− 0.063 (0.043)	7.618*(3.754)	0.993 (0.972–1.015)	4.729 (0.500–44.693)
Years of education	− 0.153**(0.037)	2.287**(0.615)	−0.118**(0.036)	− 0.577 (0.713)	0.964**(0.944–0.983)	1.093 (0.775–1.542)
Years of education^2^/100	0.101**(0.036)	− 0.974**(0.306)	0.094**(0.035)	0.234 (0.336)	1.022*(1.001–1.045)	0.933 (0.771–1.130)
Current worker	− 0.024**(0.008)	0.015 (0.025)	0.002 (0.009)	0.006 (0.028)	0.996 (0.990–1.002)	1.009 (0.989–1.029)
Longest occupation (Ref. Professional)						
Clerical	− 0.005 (0.006)	0.010 (0.027)	− 0.005 (0.006)	− 0.018 (0.026)	0.999 (0.994–1.004)	0.979 (0.951–1.008)
Manual	0.018 (0.013)	−0.009 (0.029)	0.022#(0.013)	0.029 (0.028)	1.007 (0.996–1.019)	0.986 (0.960–1.012)
Agriculture/forestry/fishery	0.011*(0.005)	0.002 (0.019)	0.003 (0.005)	−0.024 (0.019)	1.003 (0.999–1.007)	0.987 (0.971–1.003)
Domestic worker	−0.019 (0.025)	− 0.008 (0.031)	−0.059*(0.025)	− 0.007 (0.029)	0.979*(0.959–0.999)	0.981 (0.946–1.016)
Single	−0.042**(0.015)	0.026 (0.020)	−0.008 (0.015)	− 0.022 (0.017)	0.991 (0.981–1.002)	0.993 (0.980–1.006)
Home ownership	−0.001 (0.002)	− 0.076 (0.085)	−0.002 (0.002)	− 0.052 (0.096)	0.999 (0.998–1.001)	0.964 (0.902–1.030)
Group activity	−0.001 (0.002)	− 0.045 (0.044)	−0.005*(0.002)	− 0.011 (0.049)	0.999#(0.997–1.000)	0.972 (0.937–1.008)
Current smoker	0.003 (0.015)	−0.006 (0.016)	− 0.008 (0.017)	−0.010 (0.017)	0.999 (0.987–1.010)	0.996 (0.984–1.008)
Alcohol consumption	0.017 (0.015)	−0.011 (0.030)	− 0.035*(0.016)	0.015 (0.032)	0.993 (0.982–1.004)	0.999 (0.978–1.021)
Exercise	−0.004#(0.002)	− 0.018 (0.031)	−0.001 (0.002)	− 0.023 (0.034)	0.999 (0.998–1.000)	1.002 (0.980–1.025)
Hearing impairment	0.002 (0.002)	0.000 (0.008)	0.001 (0.002)	− 0.019*(0.009)	1.001 (1.000–1.002)	0.998 (0.994–1.002)
Diabetes	−0.001 (0.002)	0.012 (0.008)	−0.002 (0.003)	− 0.005 (0.010)	1.000 (0.999–1.002)	1.002 (0.995–1.008)
Hypertension	0.005*(0.002)	0.009 (0.019)	0.000 (0.002)	0.004 (0.021)	1.000 (0.999–1.002)	0.996 (0.982–1.010)
Stroke	−0.000 (0.001)	− 0.008 (0.008)	0.001 (0.002)	0.007 (0.008)	1.000 (1.000–1.001)	0.999 (0.994–1.004)
Constant		−2.753 (3.980)		8.740*(4.059)		7.189 (0.550–94.038)
Observations	3581					

^a^ Coefficients with robust standard errors in parentheses estimated by the linear model for ‘cognitive functioning’ and ‘cognitive decline’; Odds ratios with 95% confidence intervals based on robust standard errors in parentheses estimated by the logit model for ‘Cognitive impairment’. ** *p*< 0.01, * *p*< 0.05, # *p*< 0.10

^b^ Participating waves are included in the models

^c^ Cognitive impairment: Three or more incorrect answers out of nine

^d^ Analyses were conducted by STATA command ‘oaxaca’ [46]

The largest factor which contributed to gender differences in cognitive functioning at baseline was years of education (coefficient [*β*]: − 0.153, robust standard error [SE]: 0.037) while its non-linear relationship with cognitive functioning was found (*β*: 0.101, SE: 0.036). This means that women’s shorter duration of education than men accounted for women’s lower cognitive functioning on the basis of the population mean. Women’s higher proportions of being single (*β*: -0.042, SE: 0.015) and the lower proportion of them as current workers (*β*: -0.024, SE: 0.008) accounted for their lower cognitive functioning than men. However, the different proportions between men and women of the longest occupation being in agriculture/forestry/fishery (*β*: 0.011, SE: 0.005), and with hypertension (*β*: 0.005, SE: 0.002) accounted for the increased gender differences in cognitive functioning.

Although overall gender differences in cognitive decline were not found to be significant, decomposition analysis described the contributions of each endowment which can offset each other to the outcome. The educational attainment (*β*: -0.118, SE: 0.036), the proportions of the longest occupation being domestic worker (*β*: -0.059, SE: 0.025), and who were engaged with group activities (*β*: -0.005, SE: 0.002) in women contributed to women’s steeper cognitive decline than men.

Furthermore, women had a higher risk of cognitive impairment than men after three-year follow-up (odds ratio [OR] of women: 1.137, 95% confidence intervals obtained by robust standard error [CI]: 1.121–1.153; OR of men: 1.104, 95% CI: 1.088–1.121), and this difference was explained by differences in independent variables, similar to cognitive functioning and cognitive decline. Higher cognitive impairment risk among women than men was explained by education (OR: 0.964, 95% CI: 0.944–0.983) and their longest occupation was domestic worker (OR: 0.979, 95% CI: 0.959–0.999).

### Robustness checks

We performed four additional analyses for robustness checks of our findings. First, analyses using the SPMSQ without the ‘mother’s maiden name’ item were conducted (Additional file 1). The analyses for both outcomes of cognitive functioning and cognitive decline confirmed similar trends to the results with all nine items. As for cognitive functioning at baseline, educational level (*β*: -0.131, SE: 0.033), proportion who are current workers (*β*: -0.020, SE: 0.007), proportion of single individuals (*β*: -0.034, SE: 0.014), and exercise habit (*β*: -0.004, SE: 0.002) accounted for worse cognitive outcomes among women than men. On the other hand, the longest occupation being agriculture/forestry/fishery (*β*: 0.011, SE: 0.002) and the presence of hypertension (*β*: 0.005, SE: 0.002) in women had the opposite effect. As for cognitive decline, educational level (*β*: -0.094, SE: 0.033) and longest occupation being that of domestic worker (*β*: -0.046, SE: 0.023) accounted for women’s steeper cognitive decline.

Second, in the NSJE, reasons (including dementia) for why a participant was not able to complete a survey by themselves (i.e. when the proxy survey or missing was obtained) were recorded, and they partly covered the onset of cognitive impairment among non-responders. For the sake of robustness check, these reasons (hereafter referred to as ‘dementia’: *n* = 52) were included as the case ascertainment of cognitive impairment in cooperation with ‘mild decline’. Furthermore, we performed additional analyses using a different cut-off point for cognitive impairment—five or more incorrect answers out of nine questions (moderate cognitive impairment) [47]. Additional file 1 shows the results of analyses regarding cognitive impairment using three different outcomes: moderate decline, mild decline with dementia, and moderate decline with dementia. For all three outcomes, endowments mattered to women’s higher risk of cognitive impairment, and educational differences between men and women consistently contributed to gender differences in cognitive impairment, particularly for dementia in addition to mild decline (OR: 0.958, 95% CI: 0.937–0.980).

Third, to address a potential bias owing to loss to follow-up, we adopted multiple imputation method with the assumption of missing at random to impute cognitive functioning at the second wave of non-responders (*n*=443) and proxy survey (*n*=207). We decomposed factors accounted for cognitive impairment comprise mild decline and dementia, and confirmed similar results in Additional file 1: education (OR: 0.963, 95% CI: 0.941–0.985) and the longest occupation being domestic worker (OR: 0.978, 95% CI: 0.957–0.999) accounted for the women’s higher risk of cognitive impairment.

Finally, we conducted a multinominal logistic regression analysis with outcomes of non-response at the second follow-up survey and death before the second follow-up to assess the potential effects on gender differences in cognitive functioning (Table 5). Although the probability of being non-responders at the follow-up survey was similar between men and women, female respondents were 4% (95% CI: − 0.055 to − 0.027) less likely to die than male counterparts, which imply that women’s longer life expectancy could have contributed to their worse cognitive outcome.

**Table 5:** Sex differences in survey attendance and mortality in the second follow-up^a, b, c, d^

	Non-responders	Dead
Female	0.014	−0.041**
	[−0.004 to 0.031]	[−0.055 to − 0.027]
Observations	4745	4745

^a^ Marginal effects with 95% confidence intervals based on robust standard errors in parentheses estimated by multinominal logistic regression with a reference outcome of continuous attendance including proxy survey at the second follow-up survey

^b^ ** *p*< 0.01, * *p*< 0.05, # *p*< 0.10

^c^ Controlled for age, the square of age, and participating waves

^d^ Those with moderate cognitive decline (five or more incorrect answers) at baseline were excluded from the analysis (*n*=124)

## Discussion

This study aimed to investigate factors which account for gender differences in cognitive functioning in older Japanese adults by utilising the Blinder–Oaxaca method of decomposition analysis. To our knowledge, this study was the first to have added empirical evidence regarding gender differences in cognitive functioning among older Japanese adults using detailed decomposition.

Based on our analysis, the main finding of this study was that social factors related to cognitive reserve (i.e. education and occupation) account for gender differences in cognitive functioning. Lower educational attainment in women, compared to that in men, contributed to lower cognitive functioning at baseline, larger cognitive decline through follow-up, and a higher risk of cognitive impairment among women. Furthermore, the larger proportion of those solely engaged in domestic work among women also accounted for women’s worse cognitive outcomes.

In Japan, gender roles long remained static, restricting women’s educational and occupational opportunities outside the home until the middle of the nineteenth century [59]. Even after policy changes to promote women’s education, the number of women who enrolled in secondary or higher education was strikingly limited. In the early twentieth century, during which the majority of the NSJE respondent spent their lives, the idea of the ‘dutiful wife and devoted mother’, which hindered women’s participation in higher education and professional occupations by keeping them in domestic work, was proposed as the basic principle by the advisory committee of the Japanese government on educational policy. However, after the development of gender equality policies in education following the Second World War, the number of women participating in higher education began increasing, so that by 2019, the advancement rate at university and junior college was 51.6% for men and 57.8% for women [60]. Although gender inequality in occupations (e.g. labour force participation) still exist in Japan, the situation has apparently improved since the last century [61].

From the viewpoint of the cognitive reserve hypothesis, enhanced gender equality may contribute to narrowing gender disparities in dementia. Previous studies suggested that women’s health tends to be better in counties with policies supporting gender equality [62, 63]. Previous studies reported that the dementia incidence is decreasing in high income countries, particularly in Western countries, where people are potentially more engaged in intellectual activities through education and occupation [64]. Furthermore, women’s increasing active engagement in group activities and interactions with others in recent years may reduce this gender gap [65]. Further research is still needed to investigate the national prevalence of dementia and its trajectory, with factors which affect cohort differences in incidence and prevalence rates.

The current paper uniquely investigated factors which account for gender differences in cognitive functioning by using an econometric method to decompose the gender differences. Nevertheless, there were several limitations that require caution in the interpretations of our findings.

First, case ascertainment of cognitive decline was based on the SPMSQ, which was originally introduced as a screening tool. A previous validation study proved that the SPMSQ was sensitive and specific, with a sensitivity of 86.2% and a specificity of 99.0% among medical inpatients, and 66.7 and 100%, respectively, among a community sample [66]. Nevertheless, this means that neither Alzheimer’s disease nor other types of dementia, but only gross cognitive functioning or its decline, were detected. Moreover, the SPMSQ contains quite basic and simplistic items which may detect only limited aspects of domains of cognition. It is ideal to identify the detailed cognitive domains and the onset of dementia more accurately by clinical diagnosis via further investigation.

Second, our data was from a relatively small sample size, which made it impossible to make sub-group analyses by detailed age groups although we included age as a factor in our decomposition analysis. Thus, we were unable to assess whether different patterns were observed in different age groups (e.g. young–old versus old–old). Some differences might be found as the prevalence of dementia drastically increases as people get older [7].

Third, although we treated health-related variables as time-varying factors, we were unable to consider health behaviours in earlier life stages, such as childhood and adulthood as well as other unmeasured confounding factors (e.g. biological factors). Although cardiovascular morbidities tend to emerge as these behavioural risks accumulate, a particular caveat against the effects of unmeasured confounders on our analyses is necessary.

Fourth, in many population studies or datasets, even if such study or data comes from a national registry, non-responders may lead to selection bias. People’s ability to respond to and their attitudes towards a survey may affect participation; therefore, those with cognitive decline may be unable or more reluctant to join. A previous study found that the prevalence of mild cognitive impairment was higher in delayed-responders than quick-responders [67]. In the current study, we tried to deal with this potential bias by using information that respondents were not able to complete a survey by themselves due to cognitive dysfunction as the case ascertainment and by multiple imputation method, although it still may not be sufficient to cover all cases of cognitive decline among non-responders. Moreover, we found that women’s probability of death before the follow-up survey was lower than that of men, which may indicate that women’s longer life expectancy could explain observed gender differences in cognitive functioning. Men may experience cognitive decline during the survey interval before death; however, we were not able to detect this in our study. All these issues should be incorporated for a further investigation regarding gender differences in cognitive decline.

Establishing a national registry may be useful to address the limitations listed above by connecting healthcare records with other relevant information; however, challenges still exist to grasp the whole picture of dementia in the population, since dementia may be underdiagnosed [68]. Thus, improvements in knowledge regarding dementia, access to timely diagnosis, support, as well as the construction of a database regarding dementia, would be effective for obtaining better-quality evidence and the evaluation whether enhanced gender equality contributes to narrowing gender disparities in dementia, which will enhance the quality of life of those with dementia and their families, through the development of better policies.

## Conclusions

In conclusion, this study suggested that gender differences in cognitive functioning, decline, or impairment account for individually different attributes. Particularly, men seem to be more engaged in educational and occupational activities which accumulate intellectual experiences, which corresponded to the cognitive reserve hypothesis. Gender disparities in health may be partly mitigated, and prevention of cognitive decline can be promoted, by approaches to social determinants of health throughout one’s life-course, such as educational and occupational policies.

## Supplementary Information

**Additional file 1.**

## Data Availability

We used data collected by our research team (the Research Team for Social Participation and Community Health, Tokyo Metropolitan Institute of Gerontology). Data from wave1 to wave7 are publicly available from the Social Science Japan Data Archive, Centre for Social Research and Data Archives, Institute of Social Science, University of Tokyo. Although data in wave8 are not currently publicly available, they may become so in the near future.

## Abbreviations

OECD: Organisation for Economic Co-operation and Development
GDP: Gross Domestic Product
NSJE: National Survey of the Japanese Elderly
SPMSQ: Short Portable Mental Status Questionnaire
OR: Odds Ratio
SE: Standard Error
SHR: Sub-hazard ratio
CI: Confidence Interval
