# Supplementary material for: Decomposition of gender differences in cognitive functioning: National Survey of the Japanese elderly
Source: BMC Geriatr. 2021 Jan 10;21:38. doi: 10.1186/s12877-020-01990-1 (PMC7798327; doi:10.1186/s12877-020-01990-1)
Supplement: Supplementary file 1 — Additional file 1. [file 12877_2020_1990_MOESM1_ESM.docx]

Supplementary Material for

Decomposition of Gender Differences in Cognitive Functioning:

National Survey of the Japanese Elderly

Shohei Okamoto, PhD^*1^ Erika Kobayashi, PhD^*1^ Hiroshi Murayama, RN, PHN, MPH, PhD^*1*2^ Jersey Liang, PhD^*3^ Taro Fukaya, MA^*1^ Shoji Shinkai, MD, MPH, PhD^*1*4^

^*1^Tokyo Metropolitan Institute of Gerontology, 35-2 Sakaecho, Itabashi-ku, Tokyo 173-0015, Japan

^*2^The University of Tokyo, 7-3-1 Hongo, Bunkyo-ku, Tokyo 113-8656, Japan

^*3^University of Michigan, Michigan, 1415 Washington Heights, SPH II M3007, Ann Arbor, Michigan 48109-2029, USA

^*4^ Kagawa Nutrition University, 3-9-21 Chiyoda, Sakado city, Saitama 350-0288, Japan

Corresponding author: Shohei Okamoto

ORCID: 0000-0002-8580-5291

Research Team for Social Participation and Community Health, Tokyo Metropolitan Institute of Gerontology, 35-2 Sakaecho, Itabashi-ku, Tokyo 173-0015, Japan

PHONE:+81(3)3964-3241 EXT.4258 FAX:+81(3)3579-4776

E-mail: [sokamoto@tmig.or.jp](mailto:sokamoto@tmig.or.jp)

Figure A-1.　Item response theory: difficulties of memory test items

a) ‘Misreporting’ on the vertical axis denotes an incorrect report in each item of the SPMSQ.

Table A-1. Descriptive statistics for the competing-risks regression

|  | n | time at risk (Person-month) | Survival time | | | n, Cognitive impairment | n, Death without cognitive impairment |
| --- | --- | --- | --- | --- | --- | --- | --- |
|  |  |  | 25% | 50% | 75% |  |  |
| Men | 1958 | 265,955 | 156 | 249 | 301 | 571 | 893 |
| Women | 2371 | 347,468 | 146 | 232 | 299 | 922 | 691 |
| Total | 4329 | 613,423 | 154 | 237 | 299 | 1,493 | 1,584 |

Table A-2. Decomposition of risk factors: the eight SQMSQ items

|  | Cognitive functioning:  No. of incorrect answers [t=baseline] | | Cognitive decline:  Increase in the no. of incorrect answers [t+1]-[t] | |
| --- | --- | --- | --- | --- |
| Male | 0.504(0.023) | | 0.524(0.023) | |
| Women | 0.762(0.022) | | 0.547(0.021) | |
| Difference | -0.258**(0.031) | | -0.024(0.031) | |
|  | Explained | Unexplained | Explained | Unexplained |
| Overall | -0.090**(0.033) | -0.168**(0.043) | -0.118**(0.033) | 0.094*(0.044) |
| Age | 0.037(0.036) | 0.235(7.412) | 0.055(0.042) | -11.932(7.814) |
| Age^2^/100 | -0.047(0.038) | -0.009(3.636) | -0.071(0.044) | 5.722(3.795) |
| Years of education | -0.131**(0.033) | 2.149**(0.553) | -0.094**(0.033) | -0.679(0.660) |
| Years of education^2^/100 | 0.085**(0.032) | -0.885**(0.273) | 0.070*(0.033) | 0.300(0.312) |
| Current worker | -0.020**(0.007) | 0.031(0.023) | -0.002(0.008) | -0.007(0.026) |
| Longest occupation  (Ref. Professional) |  |  |  |  |
| Clerical | -0.004(0.005) | 0.010(0.025) | -0.004(0.005) | -0.015(0.025) |
| Manual | 0.020#(0.012) | -0.011(0.027) | 0.020(0.012) | 0.024(0.026) |
| Agriculture/forestry/fishery | 0.011*(0.005) | 0.010(0.017) | 0.002(0.005) | -0.024(0.017) |
| Domestic worker | -0.015(0.023) | -0.001(0.030) | -0.046*(0.023) | -0.008(0.027) |
| Single | -0.034*(0.014) | 0.019(0.018) | -0.014(0.014) | -0.024(0.016) |
| Home ownership | 0.001(0.001) | -0.096(0.076) | -0.002(0.002) | -0.037(0.093) |
| Group activity | 0.000(0.001) | -0.042(0.040) | -0.004#(0.002) | -0.009(0.046) |
| Current smoker | 0.006(0.014) | -0.001(0.015) | -0.004(0.016) | -0.011(0.016) |
| Alcohol consumption | 0.011(0.014) | -0.029(0.027) | -0.032*(0.015) | 0.016(0.030) |
| Exercise | -0.004*(0.002) | -0.026(0.029) | -0.001(0.002) | -0.014(0.032) |
| Hearing impairment | 0.002(0.002) | -0.002(0.008) | 0.000(0.002) | -0.017*(0.008) |
| Diabetes | -0.002(0.002) | 0.009(0.007) | -0.001(0.002) | -0.005(0.010) |
| Hypertension | 0.005*(0.002) | 0.007(0.018) | -0.000(0.002) | -0.002(0.020) |
| Stroke | 0.001(0.001) | -0.006(0.007) | 0.001(0.001) | 0.006(0.008) |
| Constant |  | -1.409(3.802) |  | 6.788#(4.071) |
| Observations | 3,581 | | | |

^a^ Coefficients with robust standard errors in parentheses estimated by the linear model. ** p<0.01, * p<0.05, # p<0.10

^b^ Participating waves are included in the models.

^c^ Analyses were conducted by STATA command ‘oaxaca’[1].

Table A-3. Decomposition of risk factors: Incidences of cognitive decline (moderate decline + proxy/missing due to dementia) after the three-year follow-up

|  | Moderate decline | | Mild decline + Dementia  (From proxy/missing surveys) | | Moderate decline + Dementia  (From proxy/missing surveys) | |
| --- | --- | --- | --- | --- | --- | --- |
| Male | 1.021(1.014 - 1.028) | | 1.119(1.103 - 1.137) | | 1.036(1.027 - 1.045) | |
| Women | 1.022(1.016 - 1.029) | | 1.151(1.134 - 1.168) | | 1.037(1.029 - 1.046) | |
| Difference | 0.998(0.989 - 1.008) | | 0.973*(0.953 - 0.994) | | 0.999(0.987 - 1.011) | |
|  | Explained | Unexplained | Explained | Unexplained | Explained | Unexplained |
| Overall | 0.970*(0.950 - 0.990) | 1.030*(1.004 - 1.056) | 0.947**(0.923 - 0.973) | 1.027(0.992 - 1.064) | 0.956**(0.933 - 0.980) | 1.044**(1.014 - 1.076) |
| Age | 1.014(0.987 - 1.041) | 0.050*(0.003 - 0.812) | 1.001(0.980 - 1.023) | 0.005*(0.000 - 0.837) | 1.000(0.968 - 1.032) | 0.005**(0.000 - 0.193) |
| Age^2^/100 | 0.984(0.960 - 1.009) | 3.970*(1.093 - 14.424) | 0.993(0.973 - 1.015) | 12.047*(1.079 - 134.557) | 0.995(0.966 - 1.024) | 12.220**(2.177 - 68.578) |
| Years of education | 0.992(0.982 - 1.002) | 1.346(0.909 - 1.993) | 0.958**(0.937 - 0.980) | 1.125(0.773 - 1.638) | 0.989#(0.977 - 1.001) | 1.282(0.897 - 1.833) |
| Years of education^2^/100 | 1.003(0.992 - 1.014) | 0.884(0.731 - 1.069) | 1.026*(1.002 - 1.050) | 0.927(0.754 - 1.140) | 1.003(0.988 - 1.018) | 0.920(0.767 - 1.105) |
| Current worker | 0.996(0.991 - 1.001) | 1.004(0.992 - 1.016) | 0.994#(0.988 - 1.001) | 1.009(0.988 - 1.030) | 0.993*(0.987 - 0.999) | 1.006(0.991 - 1.020) |
| Longest occupation  (Ref. Professional) |  |  |  |  |  |  |
| Clerical | 0.998(0.995 - 1.002) | 1.003(0.987 - 1.020) | 0.997(0.992 - 1.003) | 0.982(0.952 - 1.013) | 0.996(0.992 - 1.001) | 1.006(0.983 - 1.031) |
| Manual | 1.001(0.993 - 1.010) | 1.011(0.993 - 1.029) | 1.009(0.997 - 1.021) | 0.987(0.960 - 1.015) | 1.004(0.994 - 1.015) | 1.013(0.990 - 1.036) |
| Agriculture/forestry/fishery | 1.000(0.997 - 1.003) | 1.003(0.993 - 1.013) | 1.003(0.999 - 1.007) | 0.985#(0.968 - 1.002) | 1.000(0.996 - 1.004) | 1.000(0.988 - 1.013) |
| Domestic worker | 0.993(0.977 - 1.010) | 1.012(0.990 - 1.035) | 0.974*(0.952 - 0.995) | 0.979(0.941 - 1.019) | 0.984(0.964 - 1.005) | 1.013(0.980 - 1.047) |
| Single | 0.993#(0.985 - 1.001) | 0.999(0.991 - 1.006) | 0.993(0.982 - 1.003) | 0.991(0.978 - 1.005) | 0.996(0.988 - 1.004) | 0.999(0.990 - 1.008) |
| Home ownership | 1.000(0.999 - 1.000) | 0.984(0.954 - 1.015) | 1.000(0.999 - 1.001) | 0.964(0.900 - 1.033) | 1.000(0.999 - 1.001) | 0.982(0.943 - 1.023) |
| Group activity | 1.000(0.999 - 1.000) | 0.995(0.977 - 1.013) | 0.999#(0.997 - 1.000) | 0.977(0.944 - 1.012) | 1.000(0.999 - 1.000) | 1.003(0.983 - 1.024) |
| Current smoker | 0.996(0.988 - 1.003) | 1.001(0.994 - 1.008) | 0.999(0.987 - 1.010) | 0.999(0.986 - 1.012) | 0.996(0.987 - 1.005) | 1.004(0.996 - 1.012) |
| Alcohol consumption | 0.997(0.989 - 1.005) | 0.994(0.981 - 1.008) | 0.991(0.980 - 1.003) | 0.998(0.976 - 1.021) | 0.995(0.985 - 1.004) | 0.995(0.980 - 1.009) |
| Exercise | 1.000(0.999 - 1.000) | 0.995(0.980 - 1.009) | 0.999(0.998 - 1.000) | 0.997(0.973 - 1.021) | 0.999(0.998 - 1.000) | 0.989(0.971 - 1.007) |
| Hearing impairment | 1.000(1.000 - 1.001) | 0.997#(0.994 - 1.000) | 1.001(1.000 - 1.002) | 0.997(0.992 - 1.002) | 1.001#(1.000 - 1.001) | 0.996*(0.992 - 0.999) |
| Diabetes | 1.000(0.999 - 1.001) | 0.999(0.995 - 1.002) | 1.001(0.999 - 1.002) | 1.002(0.995 - 1.009) | 1.001(0.999 - 1.002) | 0.999(0.994 - 1.003) |
| Hypertension | 1.000(0.999 - 1.001) | 1.001(0.994 - 1.009) | 1.000(0.999 - 1.002) | 0.994(0.979 - 1.009) | 1.000(0.999 - 1.001) | 0.999(0.989 - 1.008) |
| Stroke | 1.000(1.000 - 1.001) | 1.001(0.998 - 1.003) | 1.000(0.999 - 1.001) | 0.999(0.994 - 1.004) | 1.000(0.999 - 1.000) | 1.001(0.998 - 1.005) |
| Constant |  | 4.415#(0.994 - 19.602) |  | 18.121*(1.204 - 272.835) |  | 14.890**(2.167 - 102.294) |
| Observations | 3,419 | | 3,633 | | 3,471 | |

^a^ Odds ratios with 95% confidence intervals based on robust standard errors in parentheses estimated by the logit model. ** p<0.01, * p<0.05, # p<0.10

^b^ Participating waves are included in the models. Mild decline: Three or more incorrect answers out of nine; Moderate decline: five or more incorrect answers out of nine; Dementia: Record of dementia in the proxy/missing surveys.

^c^ Analyses were conducted by STATA command ‘oaxaca’[1].

Table A-4. Multiple imputation for decomposition of risk factors by: Incidences of cognitive decline (mild decline + proxy/missing due to dementia) after the three-year follow-up

|  | Mild decline + Dementia  (From proxy/missing surveys) | |
| --- | --- | --- |
| Male | 1.119(1.104 - 1.136) | |
| Women | 1.154(1.137 - 1.171) | |
| Difference | 0.970**(0.949 - 0.991) | |
|  | Explained | Unexplained |
| Overall | 0.941**(0.914 - 0.968) | 1.031(0.993 - 1.070) |
| Age | 1.003(0.974 - 1.033) | 0.016(0.000 - 2.555) |
| Age^2^/100 | 0.989(0.961 - 1.018) | 7.136(0.641 - 79.441) |
| Years of education | 0.963**(0.941 - 0.985) | 1.032(0.712 - 1.495) |
| Years of education^2^/100 | 1.020(0.995 - 1.045) | 0.973(0.793 - 1.193) |
| Current worker | 0.994#(0.988 - 1.001) | 1.009(0.988 - 1.030) |
| Longest occupation (Ref. Professional) |  |  |
| Clerical | 0.998(0.993 - 1.003) | 0.989(0.959 - 1.019) |
| Manual | 1.008(0.996 - 1.019) | 0.992(0.965 - 1.019) |
| Agriculture/forestry/fishery | 1.003(0.999 - 1.006) | 0.989(0.973 - 1.005) |
| Domestic worker | 0.978*(0.957 - 0.999) | 0.989(0.956 - 1.024) |
| Single | 0.994(0.984 - 1.004) | 0.992(0.978 - 1.006) |
| Home ownership | 1.000(0.998 - 1.001) | 0.970(0.908 - 1.037) |
| Group activity | 0.999#(0.998 - 1.000) | 0.981(0.948 - 1.015) |
| Current smoker | 0.999(0.988 - 1.010) | 0.998(0.984 - 1.012) |
| Alcohol consumption | 0.991(0.979 - 1.002) | 0.999(0.977 - 1.022) |
| Exercise | 0.999(0.998 - 1.000) | 1.000(0.976 - 1.024) |
| Hearing impairment | 1.001(1.000 - 1.002) | 0.997(0.992 - 1.002) |
| Diabetes | 1.000(0.999 - 1.002) | 1.002(0.995 - 1.010) |
| Hypertension | 1.001(0.999 - 1.002) | 0.994(0.980 - 1.009) |
| Stroke | 1.000(0.999 - 1.001) | 0.999(0.993 - 1.004) |
| Constant |  | 9.842#(0.662 - 146.301) |
| Observations | 4,280 | |

^a^ Odds ratios with 95% confidence intervals based on robust standard errors in parentheses estimated by the logit model. ** p<0.01, * p<0.05, # p<0.10

^b^ Participating waves are included in the models. Mild decline: Three or more incorrect answers out of nine; Moderate decline: five or more incorrect answers out of nine; Dementia: Record of dementia in the proxy/missing surveys.

^c^ Analyses were conducted by STATA command ‘oaxaca’[1].

**References for Supplementary Material**

1. Jann B: **The Blinder-Oaxaca decomposition for linear regression models**. *Stata Journal* 2008, **8**(4):453-479.
